# Supplementary material for: Engineering orthogonal dual transcription factors for multi-input synthetic promoters
Source: Nat Commun. 2016 Dec 16;7:13858. doi: 10.1038/ncomms13858 (PMC5171851; doi:10.1038/ncomms13858)
Supplement: Supplementary Information — Supplementary Figures and Supplementary Tables [file ncomms13858-s1.pdf]

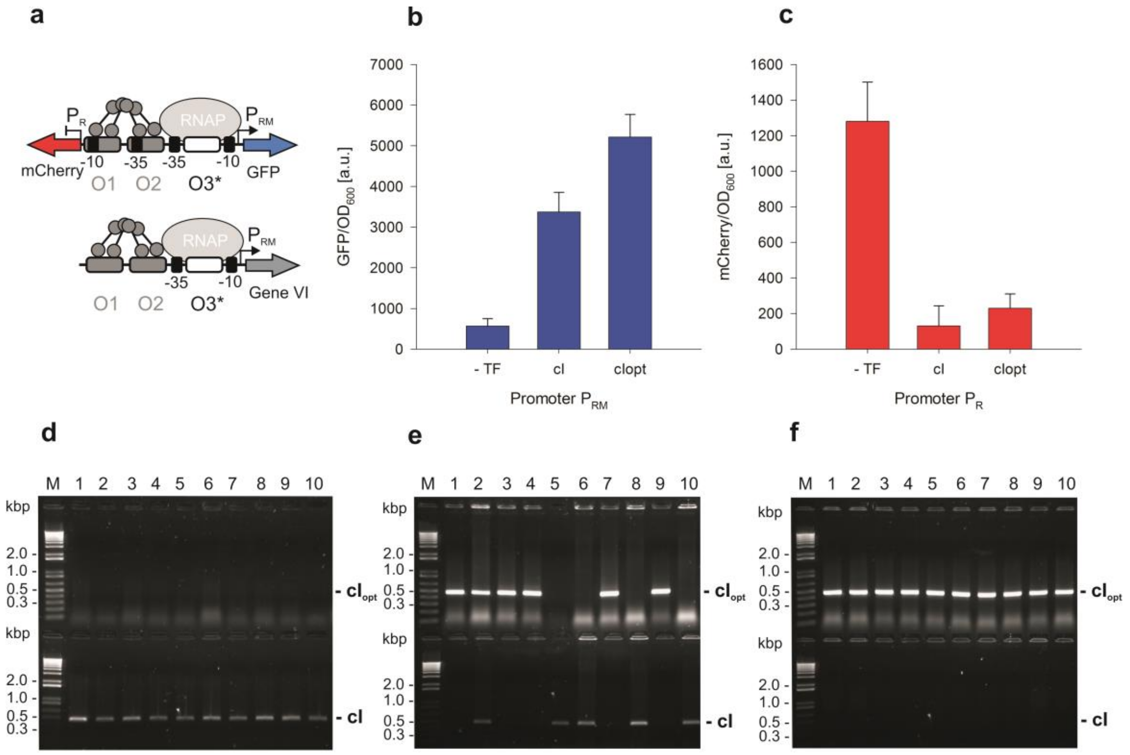

Supplementary Figure 1. Enrichment of the stronger activator $\mathbf{cI_{opt}}$ out of a $\mathbf{10^3}$-fold excess of cI-expressing phagemid. (**a**) Scheme of the λ cI binding assay with GFP and mCherry as reporter proteins (top) and the accessory plasmid with gene VI used for selection (bottom). Operator position three is obliterated to circumvent cI binding and P_{RM} repression (annotated with an asterisk). (**b**) Activation of the P_{RM} promoter used in the selection system. (**c**) Repression of the P_R promoter. Results are illustrated in the presence and absence of λ cI and cI_{opt}. GFP and mCherry expression was normalized to OD_{600} and data were obtained from four replicates. Ten colonies (1-10) were analyzed by colony-PCR after 0 rounds (**d**), 4 rounds (**e**), or 6 rounds (**f**) of selection, using the forward primer pLITMUS-F1 and the gene specific reverse primers cI_{opt}-R2 (upper gel) or cI-R3 (lower gel). Amplification of either target genes resulted in a band at ~480 bp. Cross-binding of the reverse primers between the two cI variants was initially tested and ruled out. Phage encoding cI_{opt} were fully enriched after the selection process.

O1 O2 O3

P_R/P_{RM} (natural)

GCAACCATTATCACCGCCAGAGGTAAAATAGTCAACACGCACGGTGTTAGATATTTATCCCTTGCGGTGATAGATTTAACGT
CGTTGGTAATAGTGGCGGTCTCCATTTTATCAGTTGTGCGTGCCACAATCTATAAATAGGGAACGCCACTATCTAAATTGCA

R1 **R2** **R3**

P_R/P_{RM}

GCAACCATTATCACCGCCAGAGGTAAAATAGTCAACACGCACGGTGTTAGATATTTATAAATAGTGGTGATAGATTTAACGT
CGTTGGTAATAGTGGCGGTCTCCATTTTATCAGTTGTGCGTGCCACAATCTATAAATATTTATCACCACTATCTAAATTGCA

R1 **R2** **obliterated**

$P_{CS}/P_{M,CS}$

GCAACCATTATCACCGCCGGTGATAAAATAGTCAACACCGGCGGTGATAGATATTTATAAATAGTGGTGATAGATTTAACGT
CGTTGGTAATAGTGGCGGCCACTATTTTATCAGTTGTGGCCGCCACTATCTATAAATATTTATCACCACTATCTAAATTGCA

CS **CS** **obliterated**

$P/P_{M,5C6A}$

GCAACCATTATC**CA**CGCCG**TG**GATAAAATAGTCAAC**CA**CGGCG**TG**GATAGATATTTATAAATAGTGGTGATAGATTTAACGT
CGTTGGTAATAG**GT**GCGGC**AC**CTATTTTATCAGTTG**GT**GCCGC**AC**CTATCTATAAATATTTATCACCACTATCTAAATTGCA

5C6A **5C6A** **obliterated**

$P/P_{M,5G6G}$

GCAACCATTATC**GG**CGCCG**CC**GATAAAATAGTCAAC**GG**CGGCG**CC**GATAGATATTTATAAATAGTGGTGATAGATTTAACGT
CGTTGGTAATAG**CC**GCGGC**GG**CTATTTTATCAGTTG**CC**GCCGC**GG**CTATCTATAAATATTTATCACCACTATCTAAATTGCA

5G6G **5G6G** **obliterated**

$P/P_{M,5G6T}$

GCAACCATTATC**GT**CGCCG**AC**GATAAAATAGTCAAC**GT**CGGCG**AC**GATAGATATTTATAAATAGTGGTGATAGATTTAACGT
CGTTGGTAATAG**CA**GCGGC**TG**CTATTTTATCAGTTG**CA**GCCGC**TG**CTATCTATAAATATTTATCACCACTATCTAAATTGCA

5G6T **5G6T** **obliterated**

$P/P_{M,4A5T6T}$

GCAACCATTATA**ATT**CGCCG**AAT**ATAAAATAGTCAA**ATT**CGGCG**AAT**ATAGATATTTATAAATAGTGGTGATAGATTTAACGT
CGTTGGTAATA**TAA**GCGGC**TTA**TATTTTATCAGTT**TAA**GCCGC**TTA**TATCTATAAATATTTATCACCACTATCTAAATTGCA

4A5T6T **4A5T6T** **obliterated**

$P/P_{M,4A5C6G7G}$

GCAACCATTATA**CGG**GCC**CCG**TATAAAATAGTCAA**ACGG**GGC**CCG**TATAGATATTTATAAATAGTGGTGATAGATTTAACGT
CGTTGGTAATA**TGCC**CGG**GGCA**TATTTTATCAGTT**TGCC**CCG**GGCA**TATCTATAAATATTTATCACCACTATCTAAATTGCA

4A5C6G7G **4A5C6G7G** **obliterated**

$P/P_{M,5C6A}$ For selection

GCAACCATTATC**CA**CGCCG**TG**GATAAAATAGTCAAC**CA**CGGCG**TG**GATAGATATTTATCACCGCCGGTGATAGATTTAACGT
CGTTGGTAATAG**GT**GCGGC**AC**CTATTTTATCAGTTG**GT**GCCGC**AC**CTATCTATAAATAGTGGCGGCCACTATCTAAATTGCA

5C6A **5C6A** **CS**

Supplementary Figure 2. Sequences of synthetic promoters. Synthetic promoters were derived from the consensus sequence (CS) of bacteriophage λ operators. The CS sequence was inserted into the bidirectional promoter such that the -10 and -35 regions (underlined) are unaltered. Operators are highlighted as follows: O1 blue, O2 green, O3 grey. The natural operator O3 was modified (red) in order to bypass autorepression at high cI concentrations. Mutated base pairs in O1 and O2 of synthetic promoters are bold. WT cI binding to O3 was restored for counterselections by inserting the CS, "CAACACCGGCGGTGATA" at O3 (for Fig. 4). An example is shown below ($P/P_{M,5C6A}$ For selection).

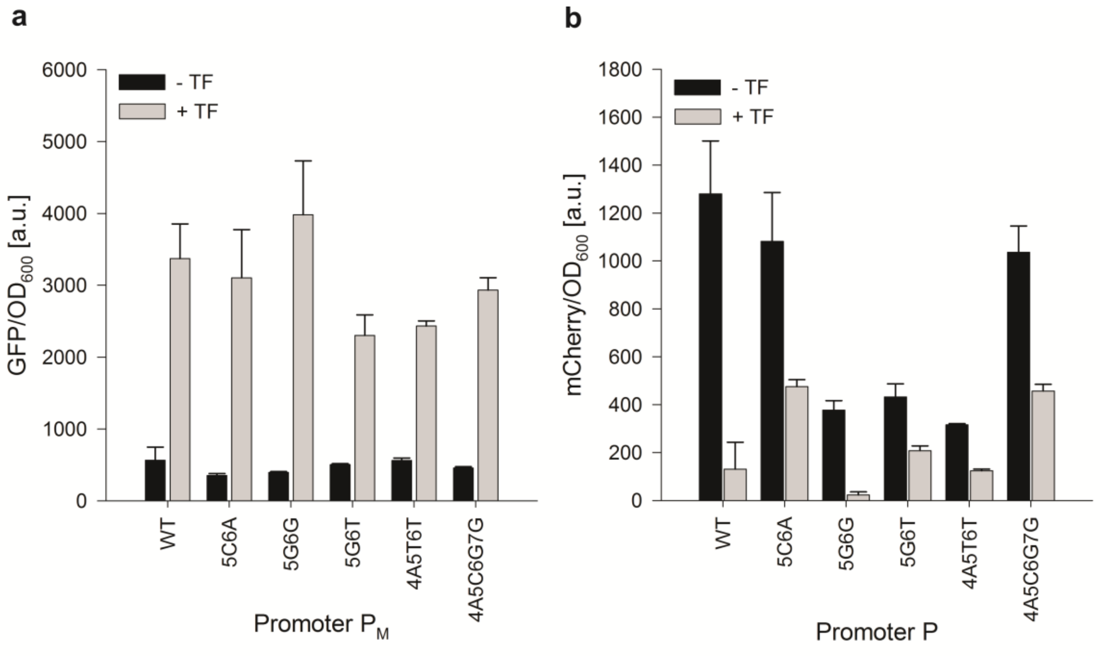

Supplementary Figure 3. Dual activation and repression of engineered promoters with phage-selected cI transcription factors (TFs). (**a**) Basal promoter strengths of the engineered promoters P_M and their activation by the selected TFs. (**b**) Basal promoter strengths of the engineered promoters P and their repression by the selected TFs presented in Fig. 4A. Results are illustrated in relation to the wild-type (WT) promoters P_{RM} and P_R in the presence and absence of λ cI. GFP and mCherry expression was normalized to OD_{600} and data were obtained from four replicates. Error bars show one standard deviation.

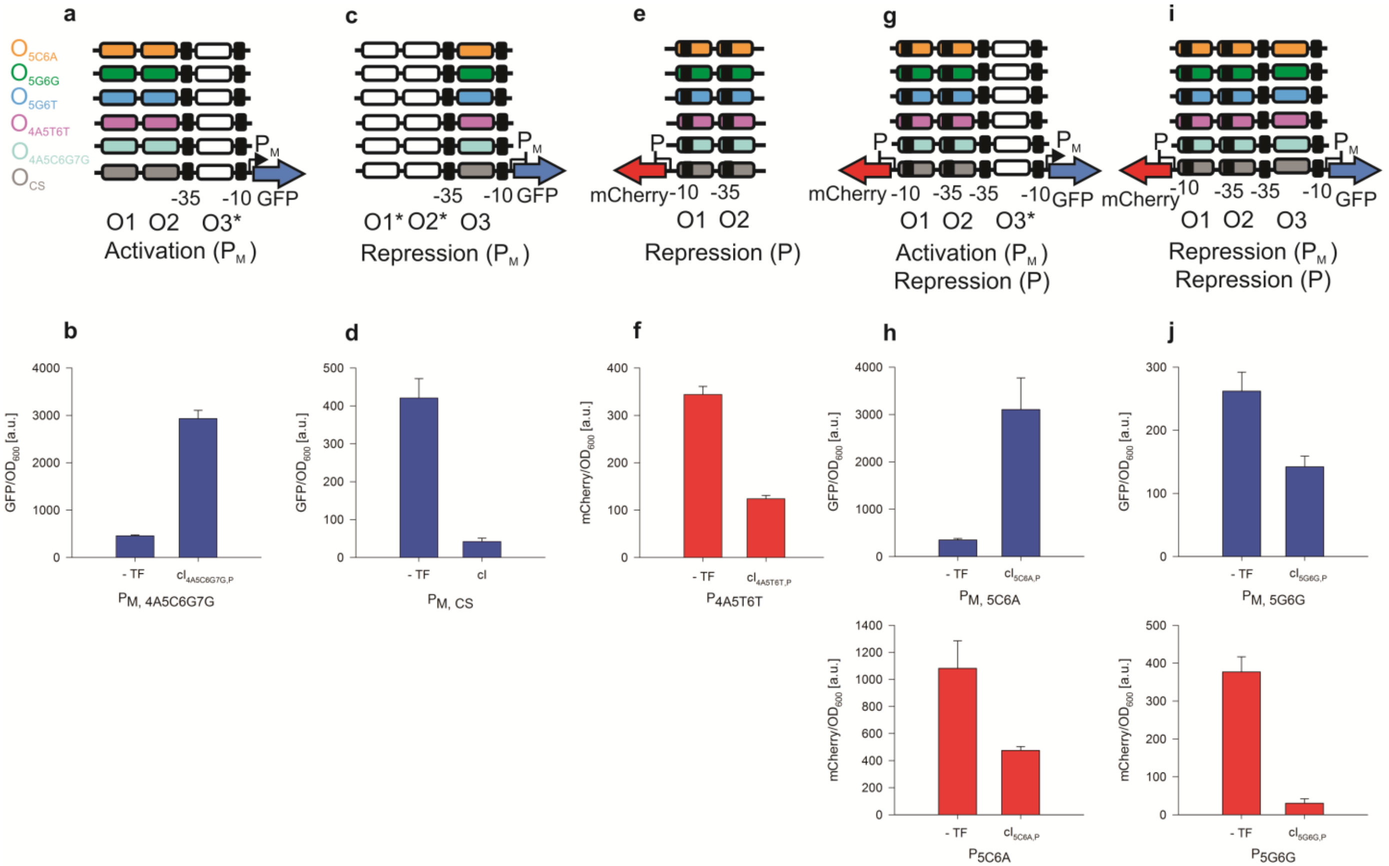

Supplementary Figure 4. Design and characterization of synthetic promoters with one input. (**a**) Set of engineered promoters P_M used for activation. (**b**) Experimental results for the activation of $P_{M,4A5C6G7G}$ by $cI_{4A5C6G7G,P}$. (**c**) Set of engineered promoters P_M used for repression. (**d**) Experimental data illustrating the repression of $P_{M,CS}$ by cI. (**e**) Set of engineered promoters P used for repression. (**f**) Experimental results depicting the repression of P_{4A5T6T} by $cI_{4A5T6T,P}$. (**g**) Set of bidirectional promoters used for dual activation-repression. (**h**) Experimental data showing the simultaneous activation of $P_{M,5C6A}$ and repression of P_{5C6A}. (**i**) Set of bidirectional promoters used for dual repression-repression. (**j**) Experimental data for the simultaneous repression of $P_{M,5G6G}$ and repression of P_{5G6G}. Operators with no cI variant binding specificity are annotated with an asterisk. Synthetic promoters P_M and P were characterized by GFP or mCherry expression, respectively. GFP and mCherry expression was normalized to OD_{600} and data were obtained from four replicates. Error bars show one standard deviation.

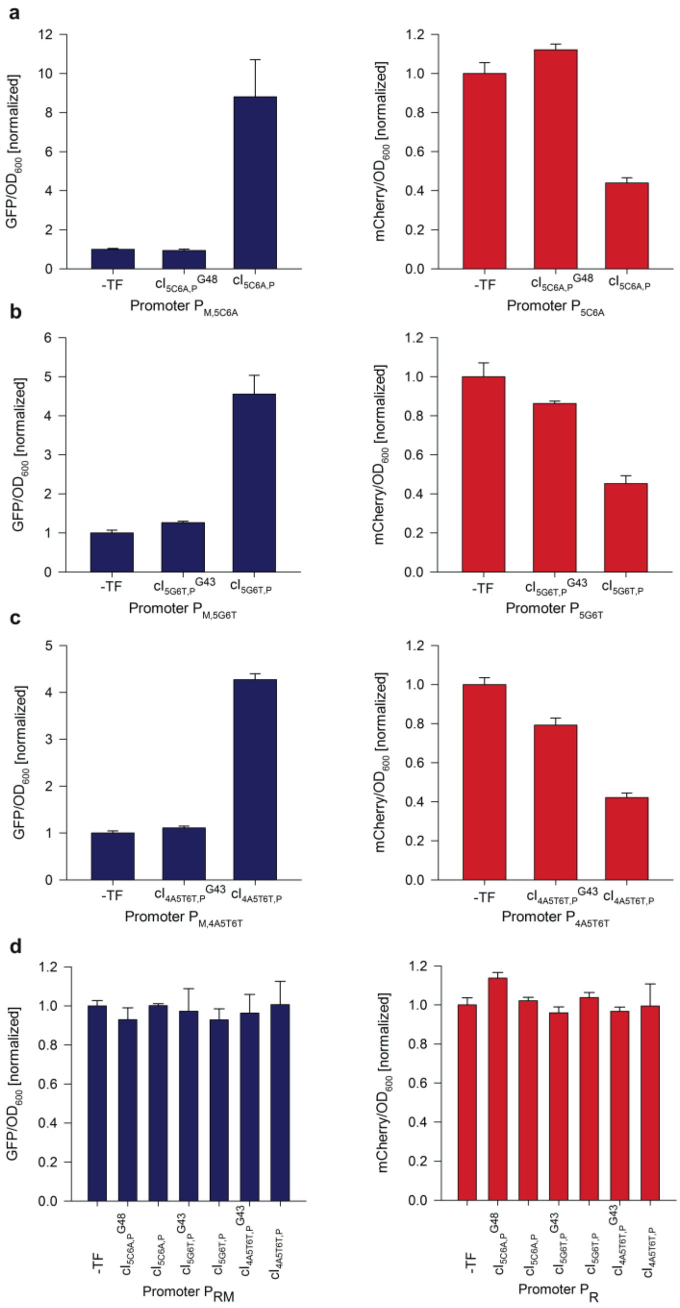

Supplementary Figure 5. Spontaneously-selected single amino acids have an impact on TF activity on engineered promoters. The effects of single amino acid mutations which were not randomized in the combinatorial libraries but were obtained during the selection process was investigated. Fold-activation of engineered λ P_M promoters and repression of synthetic λ P promoters (0.0 = 100% repression) by selected cI variants with spontaneous mutations (G43W, G48S) and their corresponding glycine variants: $cI_{5C6A,P}^{G48}$ (**a**), $cI_{5G6T,P}^{G43}$ (**b**), $cI_{4A5T6T,P}^{G43}$ (**c**). (**d**) TF activation and repression of control wild-type P_{RM} and P_R is unaffected. GFP and mCherry expression was normalized to OD_{600} and data were obtained from four replicates. Error bars show one standard deviation. Activation and repression were normalized to the basal expression of each promoter in the absence of a TF.

cI

ATGAGCACAAAAAAGAAACCATTAACACAAGAGCAGCTTGAGGACGCACGTCGCCTTAAAGCAATTTAT
GAAAAAAAGAAAAATGAACTTGGCTTATCCCAGGAATCTGTCGCAGACAAGATGGGGATGGGGCAGTCA
GGCGTTGGTGCTTTATTTAATGGCATCAATGCATTAAATGCTTATAACGCCGCATTGCTTGCAAAAATT
CTCAAAGTTAGCGTTGAAGAATTTAGCCCTTCAATCGCCAGAGAAATCTACGAGATGTATGAAGCGGTT
AGTATGCAGCCGTCACTTAGAAGTGAGTATGAGTACCCTGTTTTTTCTCATGTTCAGGCAGGGATGTTC
TCACCTGAGCTTAGAACCTTTACCAAAGGTGATGCGGAGAGATGGGTAAGCACAACCAAAAAAGCCAGT
GATTCTGCATTCTGGCTTGAGGTTGAAGGTAATTCCATGACCGCACCAACAGGCTCCAAGCCAAGCTTT
CCTGACGGAATGTTAATTCTCGTTGACCCTGAGCAGGCTGTTGAGCCAGGTGATTTCTGCATAGCCAGA
CTTGGGGGTGATGAGTTTACCTTCAAGAAACTGATCAGGGATAGCGGTCAGGTGTTTTTACAACCACTA
AACCCACAGTACCCAATGATCCCATGCAATGAGAGTTGTTCCGTTGTGGGGAAAGTTATCGCTAGTCAG
TGGCCTGAAGAGACGTTTGGCTGA

cI$_{opt}$

ATGAGCACAAAAAAGAAACCATTAACACAAGAGCAGCTTGAGGACGCACGTCGCCTTAAAGCAATTTAT
GAAAAAAAGAAAAATGAACTTGGCTTATCCCAGGAATTGGTCGCATACGAGATGGGGATGGGGCAGTCA
GGCGTTGGTGCTTTATTTAATGGCATCAATGCATTAAATGCTTATAACGCCGCATTGCTTGCAAAAATT
CTCAAAGTTAGCGTTGAAGAATTTAGCCCTTCAATCGCCAGAGAAATCTACGAGATGTATGAAGCGGTT
AGTATGCAGCCGTCACTTAGAAGTGAGTATGAGTACCCTGTTTTTTCTCATGTTCAGGCAGGGATGTTC
TCACCTGAGCTTAGAACCTTTACCAAAGGTGATGCGGAGAGATGGGTAAGCACAACCAAAAAAGCCAGT
GATTCTGCATTCTGGCTTGAGGTTGAAGGTAATTCCATGACCGCACCAACAGGCTCCAAGCCAAGCTTT
CCTGACGGAATGTTAATTCTCGTTGACCCTGAGCAGGCTGTTGAGCCAGGTGATTTCTGCATAGCCAGA
CTTGGGGGTGATGAGTTTACCTTCAAGAAACTGATCAGGGATAGCGGTCAGGTGTTTTTACAACCACTA
AACCCACAGTACCCAATGATCCCATGCAATGAGAGTTGTTCCGTTGTGGGGAAAGTTATCGCTAGTCAG
TGGCCTGAAGAGACGTTTGGCTGA

cI$_{5C6A}$

ATGAGCACAAAAAAGAAACCATTAACACAAGAGCAGCTTGAGGACGCACGTCGCCTTAAAGCAATTTAT
GAAAAAAAGAAAAATGAACTTGGCTTATCCCAGGAATCTGTCGCAGACAAGATGGGGATGGGGCAGGGG
AGGGTGAGTGCTTTATTTAATGGCATCGTGGCATTAAATGCTTATAACGCCGCATTGCTTGCAAAAATT
CTCAAAGTTAGCGTTGAAGAATTTAGCCCTTCAATCGCCAGAGAAATCTACGAGATGTATGAAGCGGTT
AGTATGCAGCCGTCACTTAGAAGTGAGTATGAGTACCCTGTTTTTTCTCATGTTCAGGCAGGGATGTTC
TCACCTGAGCTTAGAACCTTTACCAAAGGTGATGCGGAGAGATGGGTAAGCACAACCAAAAAAGCCAGT
GATTCTGCATTCTGGCTTGAGGTTGAAGGTAATTCCATGACCGCACCAACAGGCTCCAAGCCAAGCTTT
CCTGACGGAATGTTAATTCTCGTTGACCCTGAGCAGGCTGTTGAGCCAGGTGATTTCTGCATAGCCAGA
CTTGGGGGTGATGAGTTTACCTTCAAGAAACTGATCAGGGATAGCGGTCAGGTGTTTTTACAACCACTA
AACCCACAGTACCCAATGATCCCATGCAATGAGAGTTGTTCCGTTGTGGGGAAAGTTATCGCTAGTCAG
TGGCCTGAAGAGACGTTTGGCTGA

Supplementary Figure 6. Gene sequences of selected cI variants. Mutations to wild-type cI are highlighted in green and base pair substitutions to obtain the stronger activators are highlighted in blue.

cI$_{5C6A,P}$

ATGAGCACAAAAAAGAAACCATTAACACAAGAGCAGCTTGAGGACGCACGTCGCCTTAAAGCAATTTAT
GAAAAAAAGAAAAATGAACTTGGCTTATCCCAGGAATTGGTCGCATACGAGATGGGGATGGGGCAGGGG
AGGGTGAGTGCTTTATTTAATGGCATCGTGGCATTAAATGCTTATAACGCCGCATTGCTTGCAAAAATT
CTCAAAGTTAGCGTTGAAGAATTTAGCCCTTCAATCGCCAGAGAAATCTACGAGATGTATGAAGCGGTT
AGTATGCAGCCGTCACTTAGAAGTGAGTATGAGTACCCTGTTTTTTCTCATGTTCAGGCAGGGATGTTC
TCACCTGAGCTTAGAACCTTTACCAAAGGTGATGCGGAGAGATGGGTAAGCACAACCAAAAAAGCCAGT
GATTCTGCATTCTGGCTTGAGGTTGAAGGTAATTCCATGACCGCACCAACAGGCTCCAAGCCAAGCTTT
CCTGACGGAATGTTAATTCTCGTTGACCCTGAGCAGGCTGTTGAGCCAGGTGATTTCTGCATAGCCAGA
CTTGGGGGTGATGAGTTTACCTTCAAGAAACTGATCAGGGATAGCGGTCAGGTGTTTTTACAACCACTA
AACCCACAGTACCCAATGATCCCATGCAATGAGAGTTGTTCCGTTGTGGGGAAAGTTATCGCTAGTCAG
TGGCCTGAAGAGACGTTTGGCTGA

cI$_{5G6G}$

ATGAGCACAAAAAAGAAACCATTAACACAAGAGCAGCTTGAGGACGCACGTCGCCTTAAAGCAATTTAT
GAAAAAAAGAAAAATGAACTTGGCTTATCCCAGGAATCTGTCGCAGACAAGATGGGGATGGGGCAGTCC
GCGGTTTCCGAGTTATTTAATGGCATCTGGGCATTAAATGCTTATAACGCCGCATTGCTTGCAAAAATT
CTCAAAGTTAGCGTTGAAGAATTTAGCCCTTCAATCGCCAGAGAAATCTACGAGATGTATGAAGCGGTT
AGTATGCAGCCGTCACTTAGAAGTGAGTATGAGTACCCTGTTTTTTCTCATGTTCAGGCAGGGATGTTC
TCACCTGAGCTTAGAACCTTTACCAAAGGTGATGCGGAGAGATGGGTAAGCACAACCAAAAAAGCCAGT
GATTCTGCATTCTGGCTTGAGGTTGAAGGTAATTCCATGACCGCACCAACAGGCTCCAAGCCAAGCTTT
CCTGACGGAATGTTAATTCTCGTTGACCCTGAGCAGGCTGTTGAGCCAGGTGATTTCTGCATAGCCAGA
CTTGGGGGTGATGAGTTTACCTTCAAGAAACTGATCAGGGATAGCGGTCAGGTGTTTTTACAACCACTA
AACCCACAGTACCCAATGATCCCATGCAATGAGAGTTGTTCCGTTGTGGGGAAAGTTATCGCTAGTCAG
TGGCCTGAAGAAACGTTTGGCTGA

cI$_{5G6G,P}$

ATGAGCACAAAAAAGAAACCATTAACACAAGAGCAGCTTGAGGACGCACGTCGCCTTAAAGCAATTTAT
GAAAAAAAGAAAAATGAACTTGGCTTATCCCAGGAATTGGTCGCATACGAGATGGGGATGGGGCAGTCC
GCGGTTTCCGAGTTATTTAATGGCATCTGGGCATTAAATGCTTATAACGCCGCATTGCTTGCAAAAATT
CTCAAAGTTAGCGTTGAAGAATTTAGCCCTTCAATCGCCAGAGAAATCTACGAGATGTATGAAGCGGTT
AGTATGCAGCCGTCACTTAGAAGTGAGTATGAGTACCCTGTTTTTTCTCATGTTCAGGCAGGGATGTTC
TCACCTGAGCTTAGAACCTTTACCAAAGGTGATGCGGAGAGATGGGTAAGCACAACCAAAAAAGCCAGT
GATTCTGCATTCTGGCTTGAGGTTGAAGGTAATTCCATGACCGCACCAACAGGCTCCAAGCCAAGCTTT
CCTGACGGAATGTTAATTCTCGTTGACCCTGAGCAGGCTGTTGAGCCAGGTGATTTCTGCATAGCCAGA
CTTGGGGGTGATGAGTTTACCTTCAAGAAACTGATCAGGGATAGCGGTCAGGTGTTTTTACAACCACTA
AACCCACAGTACCCAATGATCCCATGCAATGAGAGTTGTTCCGTTGTGGGGAAAGTTATCGCTAGTCAG
TGGCCTGAAGAAACGTTTGGCTGA

Supplementary Figure 6 (continued). Gene sequences of selected cI variants. Mutations to wild-type cI are highlighted in green and base pair substitutions to obtain the stronger activators are highlighted in blue.

cI$_{5G6T}$

ATGAGCACAAAAAAGAAACCATTAACACAAGAGCAGCTTGAGGACGCACGTCGCCTTAAAGCAATTTAT
GAAAAAAAGAAAAATGAACTTGGCTTATCCCAGGAATCTGTCGCAGACAAGATGGGGATGTGGCAGTCC
AACTTGTCGGCTTTATTTAATGGCATCTCAGCATTAAATGCTTATAACGCCGCATTGCTTGCAAAAATT
CTCAAAGTTAGCGTTGAAGAATTTAGCCCTTCAATCGCCAGAGAAATCTACGAGATGTATGAAGCGGTT
AGTATGCAGCCGTCACTTAGAAGTGAGTATGAGTACCCTGTTTTTTCTCATGTTCAGGCAGGGATGTTC
TCACCTGAGCTTAGAACCTTTACCAAAGGTGATGCGGAGAGATGGGTAAGCACAACCAAAAAAGCCAGT
GATTCTGCATTCTGGCTTGAGGTTGAAGGTAATTCCATGACCGCACCAACAGGCTCCAAGCCAAGCTTT
CCTGACGGAATGTTAATTCTCGTTGACCCTGAGCAGGCTGTTGAGCCAGGTGATTTCTGCATAGCCAGA
CTTGGGGGTGATGAGTTTACCTTCAAGAAACTGATCAGGGATAGCGGTCAGGTGTTTTTACAACCACTA
AACCCACAGTACCCAATGATCCCATGCAATGAGAGTTGTTCCGTTGTGGGGAAAGTTATCGCTAGTCAG
TGGCCTGAAGAGACGTTTGGCTGA

cI$_{5G6T,P}$

ATGAGCACAAAAAAGAAACCATTAACACAAGAGCAGCTTGAGGACGCACGTCGCCTTAAAGCAATTTAT
GAAAAAAAGAAAAATGAACTTGGCTTATCCCAGGAATTGGTCGCATACGAGATGGGGATGTGGCAGTCC
AACTTGTCGGCTTTATTTAATGGCATCTCAGCATTAAATGCTTATAACGCCGCATTGCTTGCAAAAATT
CTCAAAGTTAGCGTTGAAGAATTTAGCCCTTCAATCGCCAGAGAAATCTACGAGATGTATGAAGCGGTT
AGTATGCAGCCGTCACTTAGAAGTGAGTATGAGTACCCTGTTTTTTCTCATGTTCAGGCAGGGATGTTC
TCACCTGAGCTTAGAACCTTTACCAAAGGTGATGCGGAGAGATGGGTAAGCACAACCAAAAAAGCCAGT
GATTCTGCATTCTGGCTTGAGGTTGAAGGTAATTCCATGACCGCACCAACAGGCTCCAAGCCAAGCTTT
CCTGACGGAATGTTAATTCTCGTTGACCCTGAGCAGGCTGTTGAGCCAGGTGATTTCTGCATAGCCAGA
CTTGGGGGTGATGAGTTTACCTTCAAGAAACTGATCAGGGATAGCGGTCAGGTGTTTTTACAACCACTA
AACCCACAGTACCCAATGATCCCATGCAATGAGAGTTGTTCCGTTGTGGGGAAAGTTATCGCTAGTCAG
TGGCCTGAAGAGACGTTTGGCTGA

cI$_{4A5T6T}$

ATGAGCACAAAAAAGAAACCATTAACACAAGAGCAGCTTGAGGACGCACGTCGCCTTAAAGCAATTTAT
GAAAAAAAGAAAAATGAACTTGGCTTATCCCAGGAATCTGTCGCAGACAAGATGGGGATGTGGCAGAAC
CGCATCTGCGCTTTATTTAATGGCATCGCGGCATTAAATGCTTATAACGCCGCATTGCTTGCAAAAATT
CTCAAAGTTAGCGTTGAAGAATTTAGCCCTTCAATCGCCAGAGAAATCTACGAGATGTATGAAGCGGTT
AGTATGCAGCCGTCACTTAGAAGTGAGTATGAGTACCCTGTTTTTTCTCATGTTCAGGCAGGGATGTTC
TCACCTGAGCTTAGAACCTTTACCAAAGGTGATGCGGAGAGATGGGTAAGCACAACCAAAAAAGCCAGT
GATTCTGCATTCTGGCTTGAGGTTGAAGGTAATTCCATGACCGCACCAACAGGCTCCAAGCCAAGCTTT
CCTGACGGAATGTTAATTCTCGTTGACCCTGAGCAGGCTGTTGAGCCAGGTGATTTCTGCATAGCCAGA
CTTGGGGGTGATGAGTTTACCTTCAAGAAACTGATCAGGGATAGCGGTCAGGTGTTTTTACAACCACTA
AACCCACAGTACCCAATGATCCCATGCAATGAGAGTTGTTCCGTTGTGGGGAAAGTTATCGCTAGTCAG
TGGCCTGAAGAAACGTTTGGCTGA

Supplementary Figure 6 (continued). Gene sequences of selected cI variants. Mutations to wild-type cI are highlighted in green and base pair substitutions to obtain the stronger activators are highlighted in blue.

$cI_{4A5T6T,P}$

ATGAGCACAAAAAAGAAACCATTAACACAAGAGCAGCTTGAGGACGCACGTCGCCTTAAAGCAATTTAT
GAAAAAAAGAAAAATGAACTTGGCTTATCCCAGGAATTGGTCGCATACGAGATGGGGATGTGGCAGAAC
CGCATCTGCGCTTTATTTAATGGCATCGCGGCATTAAATGCTTATAACGCCGCATTGCTTGCAAAAATT
CTCAAAGTTAGCGTTGAAGAATTTAGCCCTTCAATCGCCAGAGAAATCTACGAGATGTATGAAGCGGTT
AGTATGCAGCCGTCACTTAGAAGTGAGTATGAGTACCCTGTTTTTTCTCATGTTCAGGCAGGGATGTTC
TCACCTGAGCTTAGAACCTTTACCAAAGGTGATGCGGAGAGATGGGTAAGCACAACCAAAAAAGCCAGT
GATTCTGCATTCTGGCTTGAGGTTGAAGGTAATTCCATGACCGCACCAACAGGCTCCAAGCCAAGCTTT
CCTGACGGAATGTTAATTCTCGTTGACCCTGAGCAGGCTGTTGAGCCAGGTGATTTCTGCATAGCCAGA
CTTGGGGGTGATGAGTTTACCTTCAAGAAACTGATCAGGGATAGCGGTCAGGTGTTTTTACAACCACTA
AACCCACAGTACCCAATGATCCCATGCAATGAGAGTTGTTCCGTTGTGGGGAAAGTTATCGCTAGTCAG
TGGCCTGAAGAAACGTTTGGCTGA

$cI_{4A5C6G7G}$

ATGAGCACAAAAAAGAAACCATTAACACAAGAGCAGCTTGAGGACGCACGTCGCCTTAAAGCAATTTAT
GAAAAAAAGAAAAATGAACTTGGCTTATCCCAGGAATCTGTCGCAGACAAGATGGGGATGGGGCAGGCG
CGCGTTTCGGAGTTATTTAATGGCATCATGGCATTAAATGCTTATAACGCCGCATTGCTTGCAAAAATT
CTCAAAGTTAGCGTTGAAGAATTTAGCCCTTCAATCGCCAGAGAAATCTACGAGATGTATGAAGCGGTT
AGTATGCAGCCGTCACTTAGAAGTGAGTATGAGTACCCTGTTTTTTCTCATGTTCAGGCAGGGATGTTC
TCACCTGAGCTTAGAACCTTTACCAAAGGTGATGCGGAGAGATGGGTAAGCACAACCAAAAAAGCCAGT
GATTCTGCATTCTGGCTTGAGGTTGAAGGTAATTCCATGACCGCACCAACAGGCTCCAAGCCAAGCTTT
CCTGACGGAATGTTAATTCTCGTTGACCCTGAGCAGGCTGTTGAGCCAGGTGATTTCTGCATAGCCAGA
CTTGGGGGTGATGAGTTTACCTTCAAGAAACTGATCAGGGATAGCGGTCAGGTGTTTTTACAACCACTA
AACCCACAGTACCCAATGATCCCATGCAATGAGAGTTGTTCCGTTGTGGGGAAAGTTATCGCTAGTCAG
TGGCCTGAAGAAACGTTTGGCTGA

$cI_{4A5C6G7G,P}$

ATGAGCACAAAAAAGAAACCATTAACACAAGAGCAGCTTGAGGACGCACGTCGCCTTAAAGCAATTTAT
GAAAAAAAGAAAAATGAACTTGGCTTATCCCAGGAATTGGTCGCATACGAGATGGGGATGGGGCAGGCG
CGCGTTTCGGAGTTATTTAATGGCATCATGGCATTAAATGCTTATAACGCCGCATTGCTTGCAAAAATT
CTCAAAGTTAGCGTTGAAGAATTTAGCCCTTCAATCGCCAGAGAAATCTACGAGATGTATGAAGCGGTT
AGTATGCAGCCGTCACTTAGAAGTGAGTATGAGTACCCTGTTTTTTCTCATGTTCAGGCAGGGATGTTC
TCACCTGAGCTTAGAACCTTTACCAAAGGTGATGCGGAGAGATGGGTAAGCACAACCAAAAAAGCCAGT
GATTCTGCATTCTGGCTTGAGGTTGAAGGTAATTCCATGACCGCACCAACAGGCTCCAAGCCAAGCTTT
CCTGACGGAATGTTAATTCTCGTTGACCCTGAGCAGGCTGTTGAGCCAGGTGATTTCTGCATAGCCAGA
CTTGGGGGTGATGAGTTTACCTTCAAGAAACTGATCAGGGATAGCGGTCAGGTGTTTTTACAACCACTA
AACCCACAGTACCCAATGATCCCATGCAATGAGAGTTGTTCCGTTGTGGGGAAAGTTATCGCTAGTCAG
TGGCCTGAAGAAACGTTTGGCTGA

Supplementary Figure 6 (continued). Gene sequences of selected cI variants. Mutations to wild-type cI are highlighted in green and base pair substitutions to obtain the stronger activators are highlighted in blue.

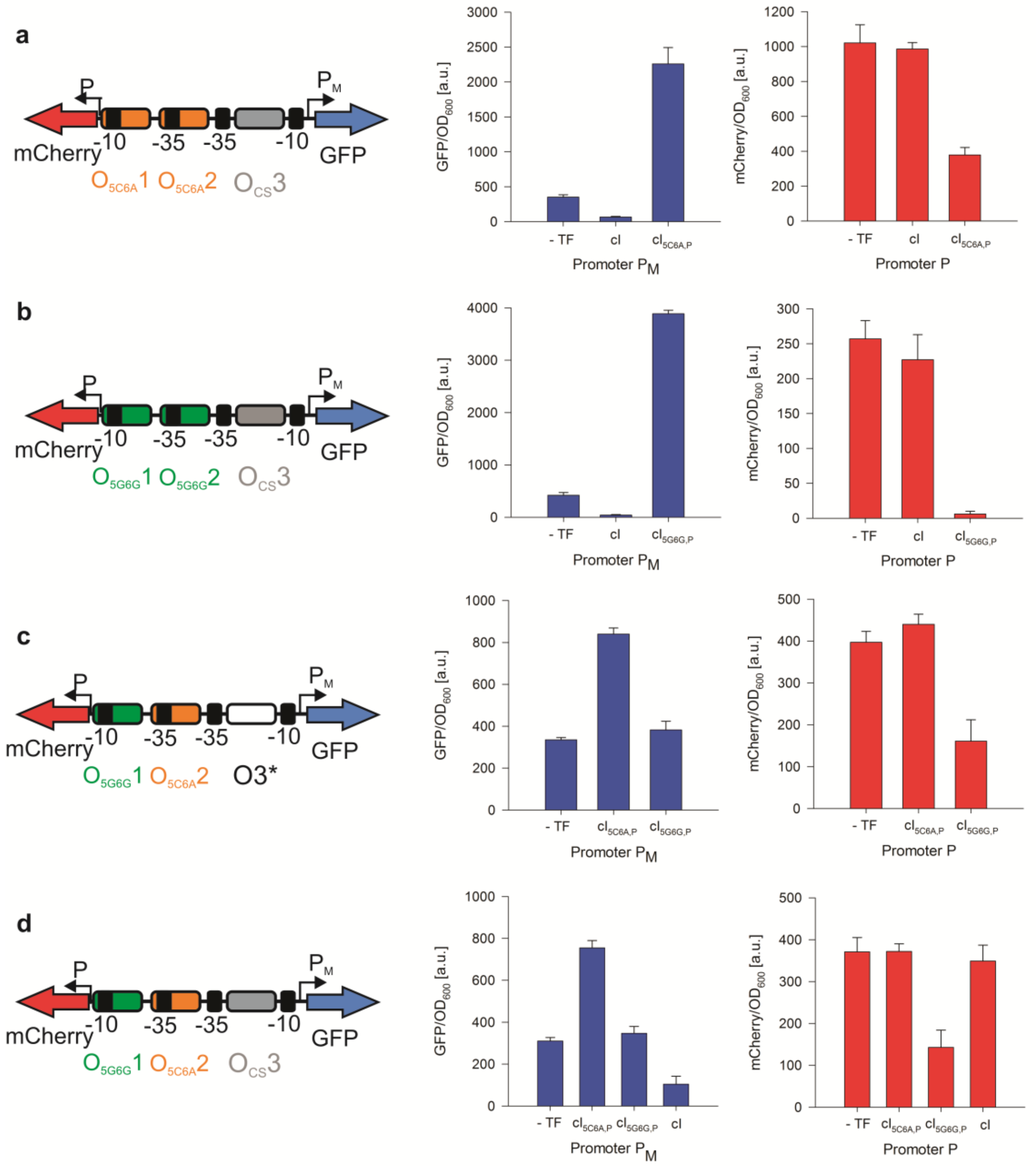

Supplementary Figure 7. Design and characterization of bidirectional promoters with two and three inputs. (**a**) Design and experimental data of a bidirectional promoter with operators $O_{5G6G}1$, $O_{5G6G}2$ and $O_{CS}3$ targeted by $cI_{5G6G,P}$ and cI. (**b**) Bidirectional promoter with operators $O_{5C6A}1$, $O_{5C6A}2$ and $O_{CS}3$ targeted by $cI_{5C6A,P}$ and cI. (**c**) Bidirectional promoter with operators $O_{5G6G}1$ and $O_{5C6A}2$ targeted by $cI_{5G6G,P}$ and $cI_{5C6A,P}$. Operator position three is obliterated to circumvent binding (annotated with an asterisk). (**d**) Bidirectional promoter with operators $O_{5G6G}1$, $O_{5C6A}2$ and $O_{CS}3$ targeted by $cI_{5G6G,P}$, $cI_{5C6A,P}$ and cI. Synthetic promoters P_M and P were characterized by GFP or mCherry expression, respectively. GFP and mCherry expression was normalized to OD_{600} and data were obtained from four replicates. Error bars show one standard deviation.

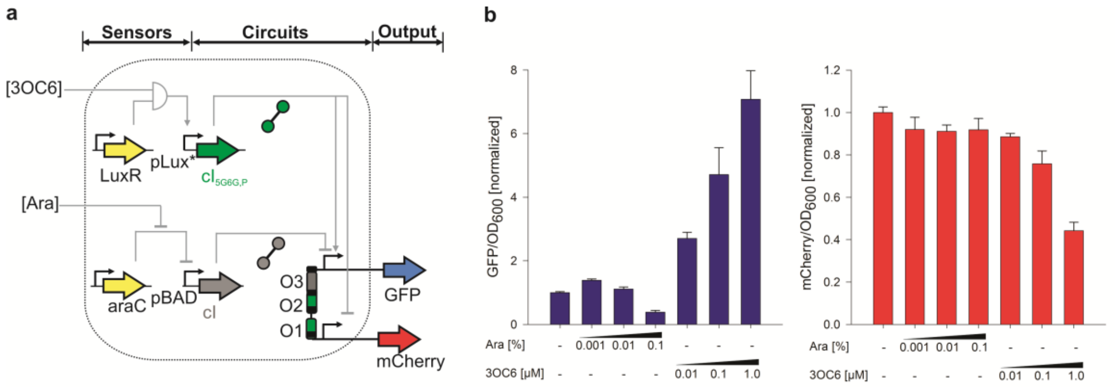

Supplementary Figure 8. Construction and characterization of a 2-input gene circuit. (**a**) Design of the 2-input gene network. Two sensors act on an integrating circuit with two cI variants (cI and $cI_{5G6G,P}$) operating on a bidirectional promoter and two reporter genes. The bidirectional promoter was designed for two inputs using the operator O_{5G6G} at position one and two and CS at position three. The expression of cI and $cI_{5G6G,P}$ was linked to supplementing arabinose and 3OC6-HSL, respectively. (**b**) Experimental data for the 2-input system illustrating the concentration-dependent response of GFP and mCherry. Addition of 3OC6-HSL resulted in a concentration-dependent increase of GFP and decrease of mCherry, whereas supplementing arabinose accounted for a concentration-dependent decrease of GFP. All data represent the average of four replicates and error bars correspond to the standard deviations between the measurements.

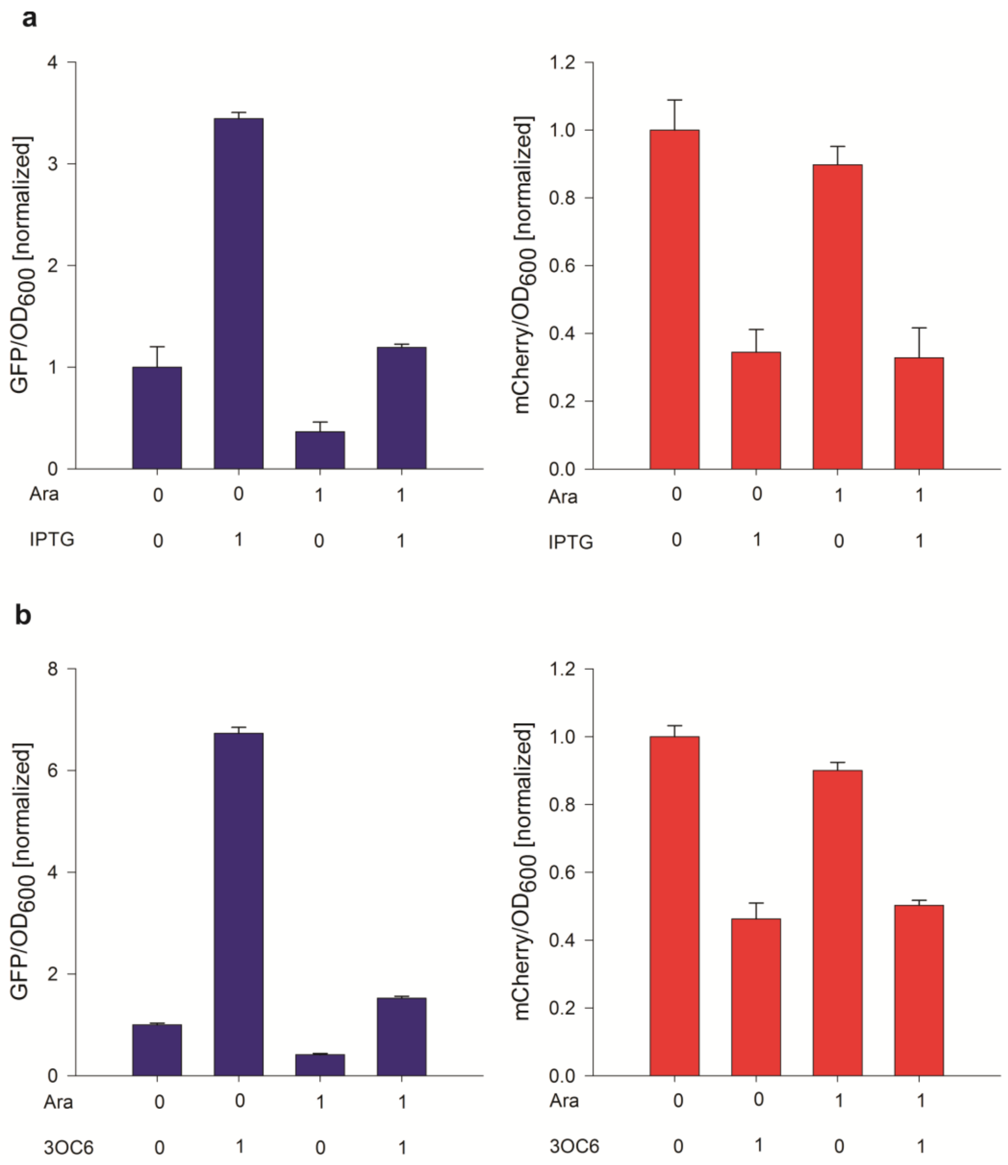

Supplementary Figure 9. Characterization of the 2-input gene circuits in binary format. (**a**) Experimental data for the gene network depicted in Fig. 5a-b with the binary inputs of 0.1% Ara and 0.1 mM IPTG used for the on state. (**b**) Experimental data for the network depicted in Supplementary Fig. 8 with the inducer concentrations of 0.1% Ara and 1.0 µM 3OC6-HSL. Activation and repression were normalized to the basal expression of each promoter in the absence of any inducer. All data represent the average of four replicates and error bars correspond to the standard deviations between the measurements.

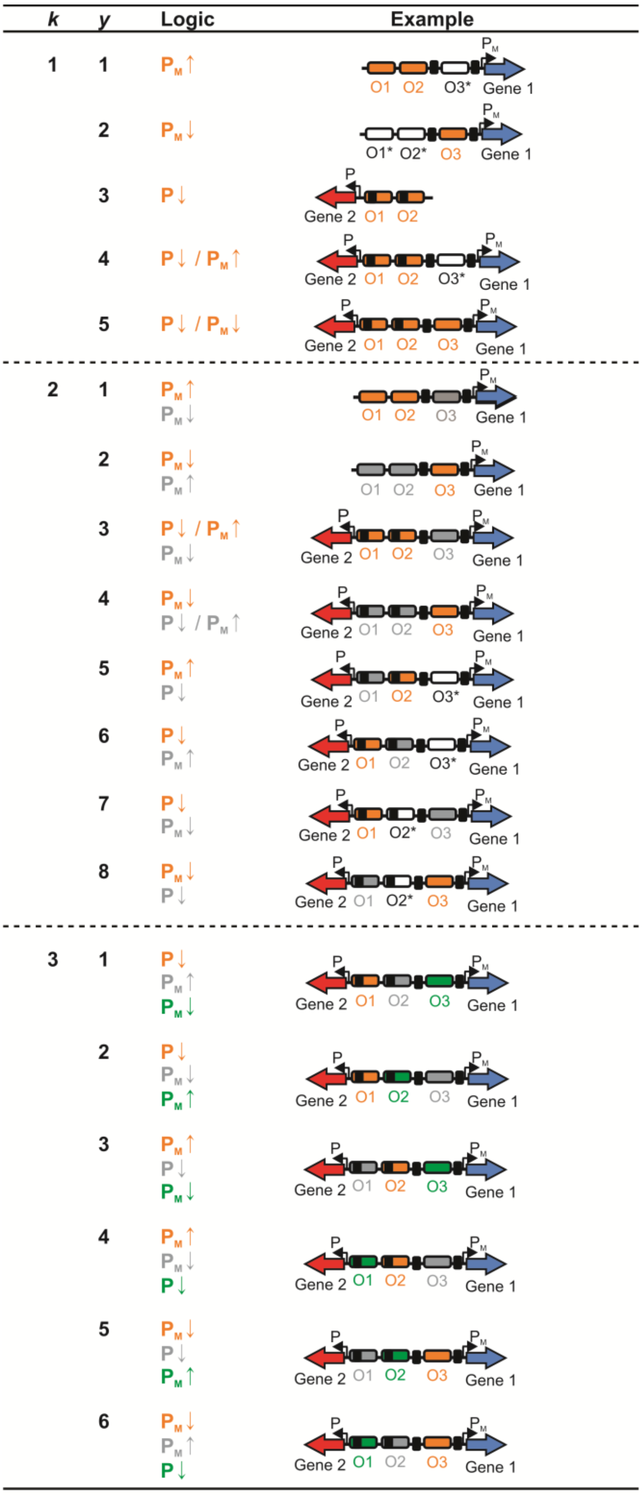

Supplementary Figure 10. Correlation between the number of inputs k and the number of logic functions y. Promoter P_M variants can get activated or repressed whereas P variants can only get repressed. This results in five ($y_{k=1}$ = 5, one input), eight ($y_{k=2}$ = 8, two inputs), or six ($y_{k=3}$ = 6, three inputs) different logics per operator combination. Promoter activation (🡅) or repression (🡇) are illustrated with arrows. Respective operators for different orthogonal cI variants are highlighted in orange, grey and green and the operator with the obliterated binding site is illustrated in white for all the exemplified promoters.

Supplementary Table 1. Combinatorial libraries used in this study.

Library	Plasmid	Randomized amino acids	Library size
Library 1	pLITMUS-rpoN-cI_{opt}-J23106-geneIII	44Q 45S 46G 47V 55N	3.2×10^6
Library 2	pLITMUS-rpoN-cI_{opt}-J23106-geneIII	45S 46G 47V 48G 55N	3.2×10^6
Library 3	pLITMUS-rpoN-cI_{opt}-J23106-geneIII	45S 46G 48G 49A 55N	3.2×10^6

Supplementary Table 2. Sequencing results of combinatorial libraries. Ten clones were sequenced from each library to confirm diversity. Wild-type amino acids are highlighted in blue, stop codons are annotated with an asterisk.

Library 1

Position	44	45	46	47	55
Clone 1	K	E	R	Y	P
Clone 2	A	R	Q	S	M
Clone 3	M	V	L	*	G
Clone 4	R	P	R	T	R
Clone 5	T	G	S	C	G
Clone 6	C	K	E	N	G
Clone 7	G	V	M	W	P
Clone 8	T	R	*	K	D
Clone 9	L	R	R	Y	N
Clone 10	G	L	E	G	P
Wild-type	Q	S	G	V	N

Library 2

Position	45	46	47	48	55
Clone 1	F	T	E	F	N
Clone 2	P	A	C	F	R
Clone 3	F	N	P	V	L
Clone 4	F	Y	L	S	M
Clone 5	G	C	L	C	A
Clone 6	I	P	M	P	T
Clone 7	F	N	P	F	N
Clone 8	S	I	G	L	Y
Clone 9	K	I	I	Y	L
Clone 10	I	T	S	I	T
Wild-type	S	G	V	G	N

Library 3

Position	45	46	48	49	55
Clone 1	S	P	P	A	P
Clone 2	T	S	G	A	N
Clone 3	A	L	L	F	F
Clone 4	S	G	G	A	N
Clone 5	L	F	L	L	F
Clone 6	F	P	L	P	A
Clone 7	K	M	R	R	N
Clone 8	A	*	M	T	N
Clone 9	S	G	G	A	N
Clone 10	no results				
Wild-type	S	G	G	A	N

Supplementary Table 3. Genotypes of *E. coli* strains used in this study.

Strain	Genotype	Company
DH5α derivative	fhuA2Δ(argF-lacZ)U169 phoA glnV44 Φ80Δ (lacZ)M15 gyrA96 recA1 relA1 endA1 thi-1 hsdR17	NEB
BL21(DE3)	fhuA2 [lon] ompT gal (λ DE3) [dcm] ΔhsdS λ DE3 = λ sBamHIo ΔEcoRI-B int::(lacI::PlacUV5::T7 gene1) i21 Δnin5	NEB
TG1	F’[traD36 lacIq Δ(lacZ) M15 proA+B+] glnV (supE) thi-1 Δ(mcrB-hsdSM)5 (rK- mK- McrB-) thi Δ(lac-proAB)	Zymo Research
TOP10	F- mcrA Δ(mrr-hsdRMS-mcrBC) Φ80lacZΔM15 Δ lacX74 recA1 araD139 Δ(araleu)7697 galU galK rpsL (StrR) endA1 nupG	Thermo Scientific

Supplementary Table 4. Plasmids used in this study.

Plasmid	Class	Antibiotic resistance	Source	Addgene ID
pLITMUS-J23106-geneIII	Phagemid	Ampicillin	This work	
pLITMUS-rpoN-cI-J23106-geneIII	Phagemid	Ampicillin	This work	80843
pLITMUS-rpoN-cI_{opt}-J23106-geneIII	Phagemid	Ampicillin	This work	80852
pLITMUS-RFP-J23106-geneIII	Phagemid	Ampicillin	This work	
pLITMUS-rpoN-cI_{5C6A}-J23106-geneIII	Phagemid	Ampicillin	This work	80905
pLITMUS-rpoN-$cI_{5C6A,P}$-J23106-geneIII	Phagemid	Ampicillin	This work	80860
pLITMUS-rpoN-$cI_{5C6A,P}^{G48}$-J23106-geneIII	Phagemid	Ampicillin	This work	
pLITMUS-rpoN-cI_{5G6G}-J23106-geneIII	Phagemid	Ampicillin	This work	80906
pLITMUS-rpoN-$cI_{5G6G,P}$-J23106-geneIII	Phagemid	Ampicillin	This work	80861
pLITMUS-rpoN-cI_{5G6T}-J23106-geneIII	Phagemid	Ampicillin	This work	80907
pLITMUS-rpoN-$cI_{5G6T,P}$-J23106-geneIII	Phagemid	Ampicillin	This work	80862
pLITMUS-rpoN-$cI_{5G6T,P}^{G43}$-J23106-geneIII	Phagemid	Ampicillin	This work	
pLITMUS-rpoN-cI_{4A5T6T}-J23106-geneIII	Phagemid	Ampicillin	This work	80908
pLITMUS-rpoN-$cI_{4A5T6T,P}$-J23106-geneIII	Phagemid	Ampicillin	This work	80863
pLITMUS-rpoN-$cI_{4A5T6T,P}^{G43}$-J23106-geneIII	Phagemid	Ampicillin	This work	
pLITMUS-rpoN-$cI_{4A5C6G7G}$-J23106-geneIII	Phagemid	Ampicillin	This work	80909
pLITMUS-rpoN-$cI_{4A5C6G7G,P}$-J23106-geneIII	Phagemid	Ampicillin	This work	80864
pLITMUS-p15A-araC-pBAD-cI-pLlac-cI_{5C6A}	Phagemid	Ampicillin	This work	
pLITMUS-p15A-araC-pBAD-cI-pLlac-cI_{5C6A}-LuxR-pLux*-$cI_{5G6G,P}$LVA	Phagemid	Ampicillin	This work	
M13KO7-ΔPS-ΔgeneIII-ΔgeneVI	Helper phage	Kanamycin	This work	80840
pJPC12-P_{RM}-B0034-geneIII	Accessory plasmid	Chloramphenicol	This work	
pJPC12-P_{RM}-B0034-geneVI	Accessory plasmid	Chloramphenicol	This work	

Supplementary Table 4 (continued). Plasmids used in this study.

Plasmid	Class	Antibiotic resistance	Source	Addgene ID
pJPC12-ΔPS-P_{RM}-B0034-geneVI	Accessory plasmid	Chloramphenicol	This work	80858
pJPC12-ΔPS-$P_{M,5C6A}$-O_{CS}3-B0034-geneVI	Accessory plasmid	Chloramphenicol	This work	
pJPC12-ΔPS-$P_{M,5G6G}$-O_{CS}3-B0034-geneVI	Accessory plasmid	Chloramphenicol	This work	
pJPC12-ΔPS-$P_{M,5G6T}$-O_{CS}3-B0034-geneVI	Accessory plasmid	Chloramphenicol	This work	
pJPC12-ΔPS-$P_{M,4A5T6T}$-O_{CS}3-B0034-geneVI	Accessory plasmid	Chloramphenicol	This work	
pJPC12-ΔPS-$P_{M,4A5C6G7G}$-O_{CS}3-B0034-geneVI	Accessory plasmid	Chloramphenicol	This work	
pJPC13-ΔPS-T7-B0034-geneVI	Accessory plasmid	Chloramphenicol	This work	
pJPC12-ΔPS-mCherry-P_R/P_{RM}-GFP	Reporter plasmid	Chloramphenicol	This work	80859
pJPC12-ΔPS-mCherry-P/$P_{M,5C6A}$-GFP	Reporter plasmid	Chloramphenicol	This work	80910
pJPC12-ΔPS-mCherry-P/$P_{M,5G6G}$-GFP	Reporter plasmid	Chloramphenicol	This work	80911
pJPC12-ΔPS-mCherry-P/$P_{M,5G6T}$-GFP	Reporter plasmid	Chloramphenicol	This work	80912
pJPC12-ΔPS-mCherry-P/$P_{M,4A5T6T}$-GFP	Reporter plasmid	Chloramphenicol	This work	80913
pJPC12-ΔPS-mCherry-P/$P_{M,4A5C6G7G}$-GFP	Reporter plasmid	Chloramphenicol	This work	80914
pJPC12-ΔPS-mCherry-O_{5C6A}1,2-O_{CS}3-GFP	Reporter plasmid	Chloramphenicol	This work	
pJPC12-ΔPS-mCherry-O_{5G6G}1,2-O_{CS}3-GFP	Reporter plasmid	Chloramphenicol	This work	
pJPC12-ΔPS-mCherry-O_{5C6A}1-O_{5G6G}2-O_{CS}3-GFP	Reporter plasmid	Chloramphenicol	This work	
pJPC12-ΔPS-mCherry-O_{5C6A}1-O_{5G6G}2-O3*-GFP	Reporter plasmid	Chloramphenicol	This work	
pJPC12-ΔPS-mCherry-O_{5G6G}1,2,3-GFP	Reporter plasmid	Chloramphenicol	This work	
pJPC12-ΔPS-O_{5G6G}1,2-O_{CS}3-mCherry-O_{5C6A}1,2-O_{CS}3-GFP	Reporter plasmid	Chloramphenicol	This work	

Supplementary Table 5. Sequencing primer used in this study.

Name	Oligonucleotide sequence
M13KO7-F1	5' GCT ACA ACG GTT AAT TTG C 3'
M13KO7-F2	5' ATG AAA AAG TCT TTA GCC 3'
M13KO7-R	5' CCA GTT ACA AAA TAA ACA GC 3'
pLITMUS-F1	5' GTC GAT TTT TGT GAT GCT CG 3'
pLITMUS-F2	5' CGT AGT TAT CTA CAC GAC G 3'
pLITMUS-F3	5' AAA AGG ATC TAG GTG AAG 3'
pLITMUS-F4	5' GCC TTT TTA CGG TTC CTG 3'
pLITMUS-R1	5' GGG TTA TTG TCT CAT GAG CGG ATA C 3'
pLITMUS-R2	5' TGC TTA TAC AAT CTT CCT G 3'
pLITMUS-R3	5' GTC AGT GCG TCC TGC TG 3'
pJPC12-F1	5' AAA CGA CGG CCA GTG AGC 3'
pJPC12-F2	5' AGC CGT ACA TGA ACT GAG 3'
pJPC12-R	5' GAT AAC AAT TTC ACA CAG G 3'

Supplementary Table 6. Oligos used for combinatorial library cloning.

Name	Oligonucleotide sequence
Library 1-F	5' GTC GCA TAC GAG ATG GGG ATG GGG NNS NNS NNS NNS GGT GCT TTA TTT AAT GGC ATC 3'
Library 1-R	5' GCG TTA TAA GCA TTT AAT GCS NNG ATG CCA TTA AAT AAA GCA CC 3'
Library 2-F	5' GTC GCA TAC GAG ATG GGG ATG GGG CAG NNS NNS NNS NNS GCT TTA TTT AAT GGC ATC 3'
Library 2-R	5' GCG TTA TAA GCA TTT AAT GCS NNG ATG CCA TTA AAT AAA GC 3'
Library 3-F	5' GTC GCA TAC GAG ATG GGG ATG GGG CAG NNS NNS GTT NNS NNS TTA TTT AAT GGC ATC NNS GCA TTA AAT GCT TAT AAC GC 3'
Library 3-R	5' GCG TTA TAA GCA TTT AAT GC 3'
